# Impact of protein kinase CK2 downregulation and inhibition on oncomir clusters 17 ~ 92 and 106b ~ 25 in prostate, breast, and head and neck cancers

**DOI:** 10.1186/s10020-024-00937-1

**Published:** 2024-10-11

**Authors:** Betsy T. Kren, Christine M. Henzler, Khalil Ahmed, Janeen H. Trembley

**Affiliations:** 1 Research Service, Minneapolis VA Health Care System, Minneapolis, MN 55417 USA; 2 Minnesota Supercomputing Institute, University of Minnesota, 117 Pleasant Street Southeast, Minneapolis, MN 55455 USA; 3 Department of Laboratory Medicine and Pathology, University of Minnesota, Minneapolis, MN 55455 USA; 4 Masonic Cancer Center, University of Minnesota, Minneapolis, MN 55455 USA; 5 Department of Urology, University of Minnesota, Minneapolis, MN 55455 USA

**Keywords:** CK2, Casein kinase 2, Oncomir, MicroRNA, Cancer, Transcription, Nanocapsule, Nanoparticle

## Abstract

**Background:**

Protein kinase CK2 is a ubiquitous and highly conserved protein Ser/Thr kinase with diverse cell functions. CK2 is upregulated in various cancers and affects numerous aspects of their underlying pathobiology. The important role of microRNAs (miRNAs) referred to as oncomirs is also recognized in various cancers. Elevation of both CK2 and altered miRNA expression in cancers raised the question whether there was a connection between CK2 function and oncomirs in cancer.

**Methods:**

PCR array analysis was used to examine the effects of CK2 siRNA-mediated downregulation on miRNA levels in C4-2 prostate cancer cells. We employed prostate cancer, breast cancer, and head and neck squamous cell carcinoma (HNSCC) cells as well as a prostate cancer xenograft orthotopic tumor model to examine the effects of CK2 siRNA-mediated downregulation or chemical inhibition on oncomir cluster miR-17 ~ 92 and miR-106b ~ 25 constituent miRNAs by quantitative reverse-transcriptase stem-loop PCR. Pri-miRNAs were measured in cancer cell lines by quantitative reverse-transcriptase PCR. Protein levels were assessed by western blot. PC3-LN4 prostate cancer orthotopic xenograft tumors and blood were collected from nude mice following repeated treatments with tenfibgen ligand nanocapsules containing RNAi-CK2 or RNAi-Control cargoes.

**Results:**

PCR array analysis demonstrated effect on a subset of miRNAs following CK2 downregulation; we focused our investigation on CK2 regulation of miR-17 ~ 92 and 106b ~ 25 oncomir clusters. Chemical inhibition or molecular downregulation of CK2 greatly reduced expression of miR-17 ~ 92 and 106b ~ 25 in prostate, breast and head and neck cancer cells in vitro. CK2α and CK2α´ protein levels were significantly correlated with many of the miR-17 ~ 92 and some of the miR-106b ~ 25 constituent members in prostate cancer cells. Decreased pri-miRNA levels for the miR-17 ~ 92 gene cluster transcript were observed for 5 of 6 cancer cell lines tested following CK2 downregulation. Nanocapsule-mediated delivery of RNAi-CK2 reduced CK2 protein expression in orthotopic prostate xenograft tumors and decreased intra-tumoral and serum levels of the oncomirs.

**Conclusions:**

Targeting CK2 for the development of new cancer therapies is under active investigation in many laboratories and pharmaceutical companies. Our data suggest a new role for CK2 in cell signaling and survival in multiple cancer types through maintenance of miR-17 ~ 92 and 106b ~ 25 biogenesis.

**Supplementary Information:**

The online version contains supplementary material available at 10.1186/s10020-024-00937-1.

## Background

The process and maintenance of oncogenesis is highly complex, involving diverse signals and pathways that overlap with the functions of protein kinase CK2 (formerly casein kinase 2 or II). CK2 is a highly conserved and ubiquitous protein S/T kinase localized in both the nuclear and cytoplasmic compartments as well as numerous intracellular sub-compartments of cells. The heterotetrameric structure of CK2 consists of two catalytic subunits (42 kDa α and 38 kDa α´) linked via two homologous regulatory subunits (28 kDa β) and forming α2β2, or αα´β2, or α´2β2 configurations depending on the cell type. The catalytic subunits are also active as monomers. CK2 appears to be constitutively active; however, a regulatory mechanism of CK2 signaling relates to its dynamic shuttling in response to altered signals (Ahmed et al. 1993; Faust et al. 1999; Trembley et al. 2023; Faust and Montenarh 2000). CK2 functions to affect gene expression at both the transcriptional and post-transcriptional levels via formation of various protein complexes and phosphorylation of numerous substrates; indeed, the broad scope of CK2 substrates in various cellular locales attests to the wide range of CK2 functions in the cell (Trembley et al. 2023; Meggio and Pinna 2003). Originally, CK2 was known to be involved in cell growth and proliferation; however, we discovered an important feature of its function that involves its role as a regulator of cell death. Elevated CK2 blocks apoptosis while downregulation of CK2 induces cell death; the latter feature provided a link to the cancer cell phenotype (Ahmed et al. 2002; Trembley et al. 2015). The essential nature of CK2 during development is indicated by failure to produce CK2α- and CK2β-knockout mouse models (Lou et al. 2008; Buchou et al. 2003). The significance of CK2 in cancer biology is suggested by its consistent elevation to varying amounts observed in different cancers, especially noted in the nuclear compartment. Considering its interaction with diverse signaling pathways in these various malignancies, it is now recognized among major players in cancer pathogenesis as documented by us and others (Tawfic et al. 2001; Trembley et al. 2009; Guerra and Issinger 2008; Borgo and Ruzzene 2019; Ruzzene and Pinna 2010).

MicroRNAs have attracted considerable attention as important molecules in cancer biology as they influence the protein translation of target mRNAs. A large number of miRNAs have been identified and evidence suggests their functional involvement in the development of diverse cancers and in other diseases. In cancer, miRNAs appear to act as tumor suppressors or oncogenes, the latter being referred to as oncomirs. Most often, miRNA function is ascribed to post-transcriptional regulation of gene expression. More recently, miRNAs are reported to play various roles in transcriptional regulation within the nucleus, among other lesser-known functions (Dragomir et al. 2022). Some patient data concerning miRNA expression are complex and not yet fully elucidated. For example, in breast cancer there are cancer sub-type and tumor grade differences in expression levels of miR-17 ~ 92 constituents in tissue and blood (Hossain et al. 2020; Jurkovicova et al. 2017; Moi et al. 2019). Because of their important biological roles, miRNAs have been considered targets for therapy development, and approaches have been developed to enhance the expression of tumor suppressor miRNAs or block the expression of oncogenic miRNAs in cancer and other diseases (Dragomir et al. 2022; Sohel 2016; Bhagirath et al. 2018; Rupaimoole and Slack 2017; Lu and Rothenberg 2018; Sekhon et al. 2016; Liu et al. 2020; Mishra et al. 2016; Bartel 2018).

MicroRNAs (miRNAs) are highly conserved short single-stranded non-coding RNA molecules of 22 nucleotides in length once fully processed. The biogenesis of miRNAs begins with RNA polymerase II-mediated transcription of the primary miRNA transcript (pri-miRNA) in the nucleus (Ha and Kim 2014). Next, the pri-miRNA is cleaved by Drosha/DGCR8 to the pre-miRNA hairpin (~ 70 nucleotides), transported to the cytoplasm by exportin 5, and further processed to a miRNA duplex by Dicer. Single-stranded mature miRNAs bound by Argonaute (Ago) proteins are loaded into RNA-induced silencing complexes (RISC) which bind to target mRNAs and typically evoke their destabilization or translational repression.

The universal upregulation of CK2 in most cancers examined emphasizes the essential involvement of CK2 in cancer biology, warranting investigations of its functional role in the pathobiology of cancer. CK2 participates in numerous biological activities in both the nucleus and cytoplasmic compartments; however, CK2 impact on post-transcriptional regulators such as microRNAs (miRNAs) in oncogenesis is poorly understood. CK2 was reported to influence miRNA levels in other studies using various anti-CK2 small molecule inhibitors. MiRNA expression was examined following CK2 inhibition using TBB in breast cancer MCF-7 cells. The authors identified 17 up-regulated and 10 down-regulated miRNAs and suggested these changes might be related to TBB suppression of cell growth (Li et al. 2014). Blocking CK2 activity using Quinalizarin inhibited adipogenesis in cultured 3T3-L1 cells, in part through up-regulation of miR-27a and miR-27b (Schwind et al. 2017). Finally, CK2 inhibition using CX-4945 modulated a subset of 6 miRNAs following injury induced by oxygen glucose deprivation in mouse optic nerve tissue (Baltan et al. 2021) These reports preliminarily suggest CK2 involvement in miRNA expression in both transformed and non-transformed cells.

We hypothesized that elevated CK2 levels contribute to cancer pathobiology in part through regulation of miRNA expression. An initial analysis in prostate cancer (PCa) cells using an array for serum- and plasma-localized miRNAs demonstrated that loss of CK2 expression caused reduced levels of certain oncomir clusters (miR-17 ~ 92 and miR-106b ~ 25). Bioinformatic analysis indicated these oncomirs were significantly elevated in TCGA prostate cancer patient samples. These paralogous oncomir clusters have been found to be dysregulated in a wide range of cancers, affecting cellular processes of tumor development, progression, and metastasis (Mehlich et al. 2018). The array results did not suggest a global impact of CK2 on miRNA biogenesis. We confirmed the array data by quantitative reverse transcriptase stem loop PCR after both CK2 molecular downregulation and enzymatic inhibition in three PCa cell lines; further, we showed that CK2 knockdown in breast cancer and head and neck cancer cells also resulted in decreased detection of these oncomirs. Reduced detection of oncomirs occurred at the pri-miRNA level as well. We validated the cultured cell data by interrogating miRNA expression levels in PCa xenograft tumors and mouse serum following malignant cell-specific RNAi-mediated targeting of CK2. The in vivo data showed similar loss of miRNA transcripts in tumor tissue and in mouse serum. Our results have revealed for the first time that CK2 supports the expression of the oncomir clusters miR-17 ~ 92 and miR-106b ~ 25 in diverse cancers, thus discovering another mechanism by which CK2 participates in oncogenic signaling.

## Methods

### Cell lines, culture and drugs

Prostate cancer LNCaP (lymph node carcinoma of the prostate) cells were obtained from ATCC (Manassas, VA, USA). Prostate cancer cells PC3-LN4 (PC3 cell line was derived from human prostate cancer bone metastasis; LN4 was derived from lymph node metastasis in mouse) and C4-2 (castration-resistant derivation of LNCaP) were obtained as described (Slaton et al. 2004; Trembley et al. 2012). PC3-LN4 cells were authenticated by Johns Hopkins University genetics core facility using a 9 marker STR profile (Baltimore, MD, USA). PC3-LN4, LNCaP, and C4-2 cells were grown in RPMI-1640 with 25 mM HEPES and l-glutamine (SH30255.01; HyClone Laboratories, Logan, UT, USA) with 10% fetal bovine serum and 1% Pen/Strep. Head and neck cancer Detroit-562 (pharyngeal tumor) and Fadu (hypopharyngeal tumor) cells were purchased from ATCC (Manassas, VA, USA) and grown in Eagle’s minimum essential medium (SH30024.01, GE Healthcare, Chicago, IL, USA) with 10% FBS and 1% penicillin/streptomycin. Breast cancer T47D (pleural effusion, infiltrating ductal carcinoma of the breast) and ZR75-1 (mammary gland tissue, ductal carcinoma) cells were purchased from ATCC and grown in RPMI-1640 with 25 mM HEPES and l-glutamine with 10% fetal bovine serum and 1% Pen/Strep. T47D cell media also contained 0.2 units/mL insulin. All cell lines were grown in monolayer culture in a 37 ºC incubator at 5% CO2. All cells had undetectable levels of mycoplasma when thawed, and were maintained in culture for no more than 2 months. TBB (also known as TBBt; 4,5,6,7-Tetrabromobenzotriazole; Adooq Bioscience, Irvine, CA, USA) was made up as a 10 mM stock in DMSO. Cells were treated with 80 μM TBB or equivalent volume of DMSO for 48 h.

### siRNA transfections

Standard chemistry siRNAs were obtained from Dharmacon (ThermoFisher Scientific). The siRNA sequences we employ targeting CK2α and α´ were validated against commercially designed siRNA sequences in a previous publication and were designed to target a highly similar nucleotide sequence between CK2α and CK2α´; there is only 1 mismatch between CK2α and CK2α´ in this sequence (Trembley et al. 2012). Thus, when we use the CK2α-specific siRNA (100% homology to CK2α), there is also knockdown of CK2α´. However, to make sure that we knockdown CK2α´ protein in cell lines which might express more CK2α´ transcript, we add in the CK2α´-specific siRNA to assure co-targeting of CK2α´.The siCK2α sense strand sequence is 5’-auacaacccaaacuccacauuu-3’. The CK2α’ sense strand sequence is 5’-auacagcccaaacuccacauuu-3’. The CK2α and CK2α’ siRNAs were co-transfected in a ratio of 3:1. The control siRNA (siControl) used was si-Non-targeting #2 (Horizon Discovery, Cambridge, UK; D-001810–02). Transfections of siRNA were performed on 60 mm plates with cells at 40 to 50% confluence with Dharmafect 1 and 2 reagents using 10 µl of Dharmafect and 20 nM siRNA total concentration.

### Immunoblot analysis

Cell pellets from cultured cells were processed in radioimmunoprecipitation assay (RIPA) buffer as previously described (Trembley et al. 2011). Tumor lysates were prepared as previously described (Ahmed et al. 2016). Fifteen to 20 µg of each lysate were subjected to electrophoresis by TGX 5–15% (18 well) midi gel system (BioRad, Hercules, CA, USA) or by Novex 4–12% Bis–Tris (20-well) midi gel system (ThermoFisher Scientific) followed by wet tank transfer to nitrocellulose membrane as recommended by the gel manufacturer. After transfer, the membranes were fully dried, rehydrated in nano-pure water, blocked for 30 min with 5% nonfat milk (Bio-Rad 170–6404) or 5% bovine serum albumin (Sigma-Aldrich Corp., Saint Louis, MO, USA, A-9647) in Tris buffered saline (TBS, pH 7.4) with 0.1% Tween 20 (TBS-T) at room temperature. Antibodies were diluted into fresh blocking buffer according to the manufacturer’s recommendations, and the membranes processed as described (Trembley et al. 2011). Antibodies used: CK2α (A300-197A; 1:3000) and CK2α΄ (A300-199A; 1:2000) from Bethyl Laboratories (Montgomery, TX, USA); CK2α’ (CSNK2A2) from ABclonal (A1616; Woburn, MA, USA; 1:1000); CK2α’ (sc-514403), CK2β (sc-46666; 1:500), Rb (sc-102; 1:2000) and Actin (sc-1616; 1:2000) from Santa Cruz Biotechnology (Santa Cruz, CA, USA); Survivin (AF886; 1:400) from R&D Systems (Minneapolis, MN, USA); E2F-1 (3742; 1:1000) and cMyc (13,987; 1:1000) from Cell Signaling Technology (Danvers, MA, USA); p53 from Epitomics (1026–1; 1:2000; acquired by AbCam; Burlingame, CA, USA). Proteins were detected by enhanced chemiluminescence (both film and using LiCor Fc imager) and quantitated as described previously (Ahmed et al. 2016; Trembley et al. 2019).

### RNA purification and quantitative reverse transcriptase PCR

Total RNA was isolated from cells or tumor using the RNeasy mini kit (Qiagen, Redwood City, Ca, USA) including on-column DNase digestion according to the manufacturer’s protocol with the following modifications. Frozen cell pellets or pulverized tumor in 1.5 mL microcentrifuge tubes were resuspended in 1 mL Trizol (Thermo Fisher Scientific, 15596018) followed by vigorous vortexing for 5–10 s and incubation at room temperature for 5 min. Next, 0.2 mL of chloroform was added with vigorous shaking for 15 s and incubation at room temperature for 3 min. Tubes were centrifuged at 4’C for 15 min at maximum microcentrifuge speed. Supernatant (0.45 mL) was transferred to a clean 1.5 mL microfuge tube followed by addition of 0.625 mL 100% ethanol and vortexing for 5–10 s. Samples (0.7 mL) were loaded into RNeasy column and further processed according to the manufacturer’s protocol. RNA was quantitated using a NanoDrop spectrophotometer.

RNA was purified from mouse serum as follows: 0.2 mL serum was added to 0.6 mL cold Trizol. All purifications were spiked with 3.5 uL [1.6 × 10^8^ copies/µL] of C. elegans miR-39 miRNA mimic (Qiagen Sciences, Germantown, MD, USA), in a 1.5 mL microfuge tube, vortexed immediately, and incubated at room temperature for 5 min. Then 0.16 mL chloroform was added and the sample vortexed and incubated at room temperature for 2–3 min. Samples were centrifuged at 4 °C in a microfuge for 20 min at 12,000*g* and the aqueous phase supernatant was immediately removed (~ 0.4 mL total volume) to a fresh microfuge tube. 1.5 × volume of 100% ethanol (nucleic acid grade) was added, the sample was vortexed and loaded onto a MinElute column placed in a collection tube (Qiagen), and centrifuged 11,000*g*, 1 min, at room temperature. The flow through was decanted and this process repeated until all supernatant/ethanol mixture was loaded on the column. The column was washed once with 0.7 mL RWT buffer with centrifugation at 11,000*g* for 1 min at room temperature followed by one wash with 0.5 mL RPE buffer and centrifugation at 11,000*g* for 1 min at room temperature. Following transfer of the column to a fresh collection tube, 0.5 mL 80% ethanol was added and the sample centrifuged at 11,000*g* for 1 min at room temperature. The MinElute column was dried by centrifuging at 14,000 rpm for 5 min with the lid open. The column was transferred to an RNase-free 1.7 mL tube with the cap cut off, 14 µL RNase free water was added and incubated ~ 3 min before centrifuging at 18,000*g* for 4 min at room temperature. RNA was pooled from 2 each 0.2 mL serum preps. RNA was quantitated using a NanoDrop spectrophotometer.

For mature miRNA cDNA synthesis reactions, poly(U) synthesis reactions were carried out as follows: RNA (1.25 μg), UTP (0.5 mM; New England Biolabs, N0450S, 1X Buffer 2 (New England Biolabs), RnaseOut (20 units; Thermo Fisher Scientific, 10777019), and poly(U) polymerase (1.5 units; New England Biolabs, M0337S) in a total volume of 12.5 μl were combined and incubated in a thermo cycler at 37 °C for 1 h. cDNA synthesis was carried out as follows: 10 μl of poly(U)-RNA was combined with 0.5 μl of universal stem loop primer (0.5 μM stock), and 0.5 μl of dNTP mix (10 mM each; Thermo Fisher Scientific, R0191) were combined, vortexed briefly, centrifuged briefly and heated at 65 °C (preheated thermocycler) for 10 min. Tubes were centrifuged briefly and 7 μl of a combined solution to final concentration of RNaseOut (2 units), DTT (10 mM) and 1X M-MLV reverse transcriptase buffer (Thermo Fisher Scientific, 18057018) was added. The tubes were tapped to mix, centrifuged briefly, and transferred to a thermocycler to equilibrate for 2 min at 37 °C. The thermocycler was paused and 1 μl [2U/uL] of M-MLV reverse transcriptase (Thermo Fisher Scientific, 28025013) added, the reaction solution mixed by pipetting, and the thermocycler restarted to incubate for 50 min at 37 °C followed by 70 °C for 15 min. Reactions were centrifuged briefly, frozen on dry ice, and stored at − 80 °C. When cDNAs were thawed, 20 uL of water was added to obtain sufficient material to run all probes, and 2.5 μl was used per PCR reaction. Thus input material represents the equivalent of 0.5 ug RNA/20 uL after dilution.

Quantitative stem-loop PCR (q-SL-PCR) was performed as described using the primers listed in Table 1 (Integrated DNA Technologies) (Ahmed et al. 2016). Each 20 μL reaction contained gene-specific miRNA forward primer or U6 forward primer (Table 1) and the universal reverse primer at the specified concentrations. Reactions were performed on an ABI 7900HT instrument FAST block with FAST SYBR green master mix (Applied Biosystems, 4385612) with the following conditions: 95 °C 10 min, 40 cycles of 95 °C 10 s and 62 °C 1 min. The primers used to detect the oncomirs in this experiment were human-specific. We tested different hybridization and extension temperatures from 58 to 62 °C using mouse and human RNA and selected 62 °C for both the hybridization and extension steps as it prevented any detection of the selected miRNAs in mouse serum using the human primers.

**Table 1:** Primers used in PCR analysis

Mature miRNA primers	Nucleotide seq 5’ to 3’
mir-17-5p fw	CGGATCAAAGTGCTTACAGTGCAG
mir-17-3p fw	CGGATACTGCAGTGAAGGCACTTG
mir-18a-5p fw	CGGTCTAAGGTGCATCTAGTGCAG
mir-19a-3p fw	CGCAGTGTGCAAATCTATGCAAAAC
mir-20a-5p fw	CTGCGTAAAGTGCTTATAGTGCAGG
mir-19b-3p fw	CGCAGTGTGCAAATCCATGCAAAAC
mir-92a-3p fw	CACAGTATTGCACTTGTCCCGGC
mir-106b-5p fw	GTCGGTAAAGTGCTGACAGTGC
mir-93-5p fw	CGTCCAAAGTGCTGTTCGTGCAG
mir-25-3p fw	CGGATCATTGCACTTGTCTCGGT
miRNA rev universal	GCAGGGTCCGAGGTATTC
Pri-miRNA primers	
pri-miR-17–92 fw	CATCTACTGCCCTAAGTGCTCCTT
pri-miR-17–92 rev	GCTTGGCTTGAATTATTGGATGA
pri-miR-25 fw	CCAGTGTTGAGAGGCGGAGACTT
pri-miR-25 rev	GGCACTGTCAGACCGAGACAAG
pri-miR-106b fw	TGCCGGGGCTAAAGTGCTGACA
pri-miR-106b rev	GCGTCCCCGTGCAAGTAACCAAG
pri-106b fw2	ATAGTGGTCCTCTCCGTGCTAC
pri-106b rev2	AACCACCCTCTCAGTGAAGG

For pri-miRNA reactions, the Superscript III cDNA Reverse Transcriptase Kit (Thermo Fisher Scientific) was used to synthesize cDNA from total RNA (1 µg input) using oligo-dT primers according to the manufacturer’s protocol and with the following conditions: 5 min 65 °C, ice for 5 min, centrifuge tube by a quick spin, 50 min 50 °C, 5 min 85 °C. Input cDNA into PCR reactions corresponded to 0.5 µg of original RNA input to the cDNA reaction. Each reaction contained forward and reverse primers listed in Table 1 using 500 nM each per 20 µL reaction. Reactions were run on a ABI 7900HT instrument FAST block with PowerUp SYBR Green Master Mix (Thermo Fisher Scientific) with the following cycling conditions: 20 s at 95 °C followed by 95 °C for 3 s and 60 °C for 30 s for 40 cycles.

Analyses were performed using the SDS 2.3 ABI software and changes calculated according to the 2(-∆∆Ct) method. RNU6-2 was used as the reference gene for tumor and serum miRNA normalization and cell derived RNA. B2M was used as the reference gene for pri-miRNAs in prostate and TBP was used as the reference gene for pri-miRNA in breast and head and neck. All results are reported as the average of reactions from 2 to 3 independent experiments for cultured cells, from 8 tumors per group, and from 4 mouse sera per group with each sample run in duplicate.

Primers used were as follows: Pre-designed primers were obtained from Integrated DNA Technologies, including normalization controls for pri-miRNAs B2M Hs.PT.58v.1875958 and TBP Hs.PT.58v.39858774. RNU6-2 miScript Primer Assay was obtained from Qiagen (MS00033740) for miRNA normalization.

### PCR array processing

RNA was purified from 3 independent C4-2 cell pellets each representing siCK2 and siControl conditions (see siRNA transfection and RNA purification sections) and used to perform PCR array analysis on the miScript™ miRNA PCR Array for Human Serum & Plasma according to the manufacturer’s protocol (Qiagen, MIHS-106Z #331221). The plate was run using Applied Biosystems models 7900HT (Fast block). Data was analyzed using the miScript miRNA PCR Data Analysis spreadsheets available from Qiagen. The data reported represents the mean fold change of siCK2 relative to siControl for 3 independent samples per condition, each sample analyzed in duplicate for each miRNA. Normalization controls included on the array were: SNORD61, SNORD68, SNORD72, SNORD95, SNORD96A, and RNU6B/RNU6-2.

### Prostate cancer xenograft experiment

CD1 nude male mice were obtained from Charles River (strain code 086). Experiments were initiated in mice at age 7–8 weeks. Orthotopic tumors were initiated by injection of 1 million PC3-LN4 cells and tumors tracked as described previously (Trembley et al. 2011). Treatments began 7 days after tumors were initiated. Tenfibgen (TBG)-RNAi-CK2 and TBG-RNAi-F7 (Control) nanocapsule preparation and tail vein injections were performed as described previously (Ahmed et al. 2016). Injections were performed every 3 days: the first injection dose was 10 mg/kg and all subsequent injections were performed at 0.1 mg/kg for a total of 10 treatments. Mice were sacrificed 1 day following the final treatment. Primary tumors were collected, measured with calipers, and weighed. Then soft dead tissue was removed by gentle scraping after slicing the tumor tissue in half and the remaining intact tumor pieces weighed. Metastatic tumors were collected and weighed. Blood serum was collected as described previously (Ahmed et al. 2016).

Mice were housed at the Minneapolis Veterans Affairs Health Care System facilities which are approved by AAALAC International in accordance with the current regulations and standards of the United States Department of Agriculture (USDA) and National Institutes of Health (NIH) Department of Health and Human Services (DHHS) (Trembley et al. 2014). Animal experimental protocols were approved to be conducted at the Minneapolis Veterans Affairs Health Care System in strict accordance with the recommendations in the Guide for the Care and Use of Laboratory Animals of the National Institutes of Health. The protocol was approved by the Minneapolis Veterans Affairs Health Care System Institutional Animal Care and Use Committee and by the University of Minnesota Institutional Animal Care and Use Committee. All surgical procedures, as approved by the above-mentioned committees, were performed under inhaled isoflurane anesthesia, and all efforts were made to minimize suffering.

Endpoint criteria with respect to orthotopic prostate tumor growth included: palpated tumor > 1.5 cm diameter; orthotopic tumor causing severely impaired mobility or palpable urinary obstruction. Mice were observed for mobility and orthotopic prostate tumors were tracked by palpation every 3 days to assess for endpoint criteria. Once palpation indicated that the longest tumor dimension was approximated as greater than 1.5 cm (5 of 16 mice), all mice were euthanized. Once removed from the intraperitoneal cavity and measurable using calipers, 7 of 8 control and 2 of 8 anti-CK2 treated tumor lengths exceeded 1.5 cm (tumor lengths ranged from 0.9 to 2.2 cm). This study was performed in accordance with the approved criteria to the best of our ability as all mice were euthanized once palpated tumors began to exceed 1.5 cm.

### Human cancer TCGA data analysis

TCGA miR-seq data was used to assess whether there was evidence for differential expression of the miR-17 ~ 92 and 106b ~ 25 oncomir members in prostate cancer samples compared to paired normal prostate tissue. For each miRNA, a paired 2-tailed t-test was used to compare log_2_(FPKM) expression between tumor and matched normal samples (N = 52). A Bonferroni correction was applied to adjust for multiple hypothesis testing.

### Human CancerMIRNome data analysis

The following dataset with associated differential expression analysis and statistical significance data was accessed via CancerMirNome: GSE112264 Li et al. 2022. GSE112264 contains serum microRNA profiles of 1591 male samples, including 809 prostate cancers, 241 negative prostate biopsies, 50 esophageal cancer, and 41 non-cancer controls.

### Statistical analysis

Most analyses were performed using Prism 6: P-values for PCR array miRNA fold change, percent dead tumor, metastatic tumor mass and miRNA expression change in tumor and serum were calculated by unpaired 2-tailed t-tests, with no correction for % dead tumor tissue and metastatic tumor mass data and Welch’s correction or Mann Whitney test depending on data distribution and number of samples for miRNA data. Relative miRNA level changes for cultured cells are represented as mean with standard error of the mean. Pearson r correlation matrix analyses were performed in Prism 10 using the respective quantitated CK2 subunit protein normalized expression levels for siCK2 and siCtrl conditions (relative to untreated samples of the same cell line) and miRNA normalized data values (relative to untreated samples of same cell line) in combined prostate cancer cell line data (N = 12 input values per analyte). The analyses were performed individually for each CK2 subunit, and p values were calculated by Prism 10 software.

## Results

### Inhibition of CK2 expression and activity decreases oncomir expression in prostate cancer cells

**Fig. 1:**
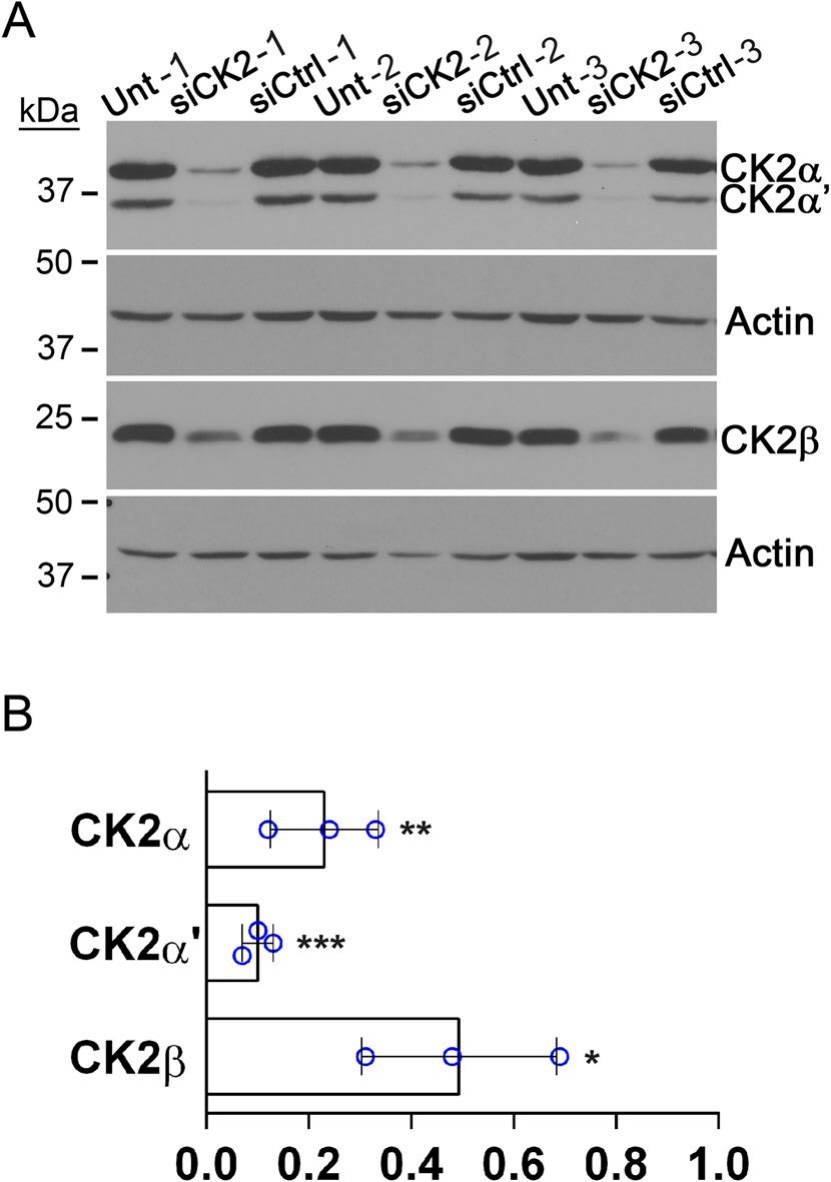
Immunoblot analysis of CK2 downregulation in C4-2 cells. **A** Immunoblot analysis following siRNA transfection (72 h). Proteins detected are indicated on the right side of the blots. Actin signal was used as the loading control. **B** Chart representing quantitation of protein signals relative to si-Ctrl (siRNA non-targeting control). Data points from 3 experimental replicates. One sample t-test, 2-tailed. *p < 0.05; **p < 0.01; ***p < 0.001. Error bars represent standard deviation. Antibodies: CK2α (A300-197A); CK2α΄ (A300-199A); CK2β (sc-46666); Actin (sc-1616)

As part of our continued investigations into cell regulatory functions for CK2 in prostate, we performed PCR array analyses for miRNAs found to be detectable and differentially expressed in human serum and plasma for various pathophysiologies. These analyses measured changes in triplicate for miRNA expression levels resulting from siRNA-mediated CK2 downregulation in human prostate cancer C4-2 cells. Non-targeting siRNA was used as a control comparison. Twenty-two miRNAs were found to be differentially regulated between control and siCK2 groups (Table S1). In particular, we identified that numerous members of the oncomir clusters miR-17 ~ 92 and miR-106b ~ 25 were significantly downregulated because of decreased CK2 protein expression (Table 2). These oncomirs are reported to demonstrate increased expression in PCa tumor tissue and cell lines (Zhou et al. 2016; Feng et al. 2017; Hsu et al. 2014; Al-Kafaji et al. 2016; Kelly et al. 2013), including in matched normal and malignant prostate tissue from The Cancer Genome Atlas (TCGA) data (Table 2). Not all miRNAs were downregulated (Table S1). For example, CK2 downregulation resulted in increased expression of miR-122-5p and miR-143-3p, miRNAs with reported lower expression levels in prostate cancer tissues relative to normal prostate (Kumar et al. 2018; Liu et al. 2019); these changes were not significant. Downregulation of CK2 subunits was confirmed by immunoblot (Fig. 1).

**Table 2:** Effect of CK2 downregulation in C4-2 cells on miRNAs found in human serum and plasma

miRNA (mature)	Fold Regulation siCK2 vs. siCtrl	p value^a^	Level in Paired Prostate Tumor vs. Normal	p value^b^
hsa-miR-17-5p	− 2.27	0.006	Up	1.15e− 15
hsa-miR-17-3p	− 1.51	0.021	Up	5.66e−16
hsa-miR-18a-5p	− 2.28	0.007	Up	1.29e−07
hsa-miR-19a-3p	− 2.29	0.030	Up	2.269e−11
hsa-miR-19b-3p	− 1.92	0.035	Up	1.91e−12
hsa-miR-20a-5p	− 1.88	0.036	Up	4.64e−17
hsa-miR-93-5p	− 1.49	0.006	Up	1.14e−18
hsa-miR-106b-5p	− 1.89	0.021	Up	9.95e−14

^a^t-test based on 2^(-delta Ct)^ values; n = 3 experimental replicates (2 technical replicates per sample) for siCK2 and siCtrl, 48 h

^b^paired t-test with Bonferroni correction using prostate cancer RNA-seq TCGA data; n = 52

**Fig. 2:**
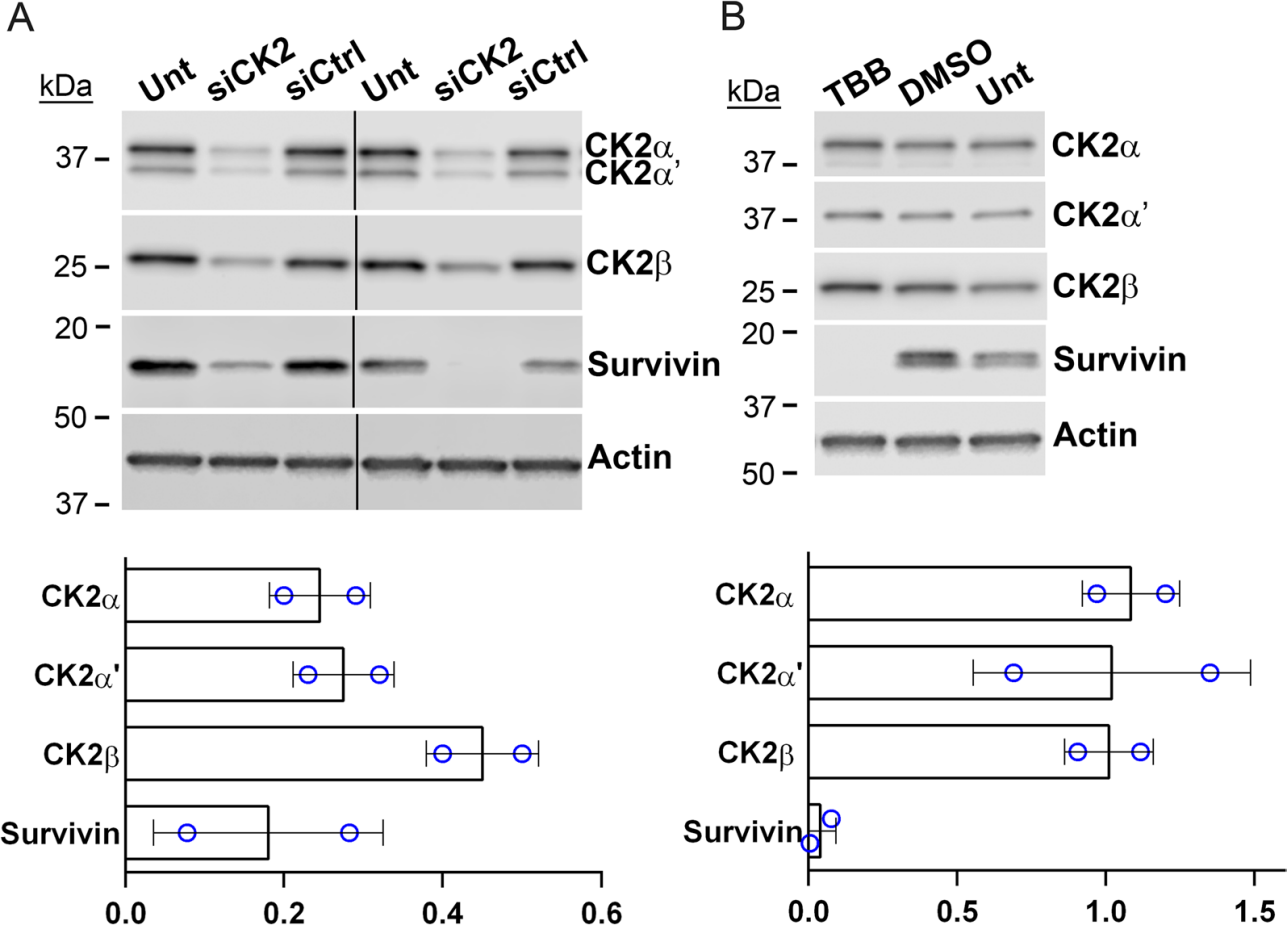
Immunoblot analysis of CK2 downregulation and chemical inhibition in PC3-LN4 cells. **A** Upper panel: Immunoblot analysis following siRNA transfection (48 h). Proteins detected are indicated on the right side of the blots. Actin signal was used as the loading control. Lower panel: Chart representing quantitation of protein signals relative to si-Ctrl treatment. Data points from 2 biological experiments. siCtrl = siRNA for non-targeting control. Antibodies: CK2α (A300-197A); CK2α΄ (A300-199A); CK2β (sc-46666); Actin (sc-1616). **B** Representative immunoblot analysis following TBB small molecule anti-CK2 inhibitor treatment (48 h). Proteins detected are indicated on the right side of the blots. Actin signal was used as the loading control. Antibodies: CK2α (A300-197A); CK2α΄ (A1616); CK2β (sc-46666); Survivin (AF886); Actin (sc-1616). Lower panel: Chart representing quantitation of protein signals relative to si-Ctrl treatment. Vertical lines indicate removal of lanes. Data points from 2 experimental replicates per cell line

Loss of miR-17 ~ 92 and miR-106b ~ 25 expression due to CK2 downregulation in C4-2 cells was confirmed by quantitative reverse-transcriptase stem loop PCR (qRT-SL-PCR) (Table 3). Two further PCa cell lines were analyzed by q-RT-SL-PCR for expression levels of miR-17 ~ 92 and miR-106b ~ 25 member miRNAs following CK2 downregulation. As shown in Table 3, oncomir miR-17 ~ 92 and miR-106b ~ 25 expression levels were greatly reduced in LNCaP and PC3-LN4 cells. Decreased CK2 expression for C4-2 and PC3-LN4 is shown in Figs. 1, 2A, respectively; evidence for CK2 protein downregulation in LNCaP (CK2α 0.54 [0.36,0.73]; CK2α' 0.43 [0.19,0.68]; CK2β 0.49 [0.35,0.62]) cells was previously published (Trembley et al. 2012).

**Table 3:** Expression of oncomir clusters in response to CK2 downregulation in PCa cells

miR-17 ~ 92							
Cell Line	17-5p	17-3p	18a-5p	19a-3p	19b-3p	20a-5p	92a-3p
C4-2	0.35 ± 0.02	0.81 ± 0.14	0.51 ± 0.17	0.28 ± 0.02	0.36 ± 0.10	0.21 ± 0.08	0.37 ± 0.10
LNCaP	0.64 ± 0.08	0.68 ± 0.00	0.75 ± 0.15	0.86 ± 0.27	0.67 ± 0.19	0.70 ± 0.12	0.75 ± 0.09
PC3-LN4	0.36 ± 0.08	0.89 ± 0.26	0.38 ± 0.08	0.44 ± 0.15	0.42 ± 0.16	0.34 ± 0.09	0.37 ± 0.02

miR-106b ~ 25							
	25-3p	93-5p	106b-3p				
C4-2	0.69 ± 0.36	0.30 ± 0.00	0.77 ± 0.3				
LNCaP	0.78 ± 0.03	0.70 ± 0.12	0.63 ± 0.14				
PC3-LN4	0.48 ± 0.07	0.42 ± 0.11	0.56 ± 0.07				

^*^ Cells were transfected with 20 nM siCK2 siRNAs; PC3-LN4, C4-2 48 h, LNCaP 72 h. Expression levels were determined using the ∆∆Cq method relative to siCtrl cells. Data shown is the mean ± 1 SEM from two experimental replicates

We next examined whether blocking catalytic activity using a CK2 small molecule inhibitor reduced miR-17 ~ 92 and miR-106b ~ 25 levels in these same cell lines. A very similar loss of oncomir member levels was observed due to CK2 inhibition with TBB (Table 4). No notable change was observed in CK2 subunit protein levels for all three prostate cancer cell lines, as shown in Fig. 2B for PC3-LN4 and previously documented for LNCaP and C4-2 (Trembley et al. 2019). The effect of TBB on CK2 activity in LNCaP and C4-2 cells was documented by immunoblot demonstrating decreased AR and NFkB p65 levels in these cells (Trembley et al. 2019. We have previously published that loss of Survivin expression results from RNAi and TBB-mediated targeting of CK2 in PCa (Wang et al. 2008). In these experiments we also observed reduced Survivin expression in siCK2-transfected and in TBB-treated PC3-LN4 cells, indicating the efficacy of CK2 inhibition in these cells (Fig. 2). In fact, CK2α and CK2α' steady-state protein levels demonstrated a significant positive correlation with survivin steady-state protein levels across these 3 prostate cancer cell lines comparing siCK2 to siControl conditions (unpublished data).

**Table 4:** Expression of oncomir clusters in response to CK2 inhibition by TBB in PCa cells

miR-17 ~ 92							
Cell Line	17-5p	17-3p	18a-5p	19a-3p	19b-3p	20a-5p	92a-3p
C4-2	0.27 ± 0.02	0.82 ± 0.18	0.28 ± 0.02	0.32 ± 0.03	0.31 ± .05	0.53 ± 0.06	0.47 ± 0.06
LNCaP	0.41 ± 0.09	0.63 ± 0.33	0.35 ± 0.07	0.31 ± 0.06	0.31 ± 0.07	0.53 ± 0.09	0.48 ± 0.13
PC3-LN4	0.52 ± .18	0.82 ± 0.11	0.43 ± 0.13	0.58 ± .19	0.55 ± 0.20	0.61 ± .21	0.89 ± 0.26

miR-106b ~ 25							
	25-3p	93-5p	106b-3p				
C4-2	0.69 ± 0.36	0.30 ± 0.00	0.77 ± 0.29				
LNCaP	0.64 ± 0.08	0.50 ± 0.03	0.42 ± 0.09				
PC3-LN4	0.48 ± 0.10	0.42 ± 0.11	0.56 ± 0.07				

^*^Cells were treated with 80 µM TBB for 48 h. Expression levels were determined using the ∆∆Cq method relative to siCtrl cells. Data shown is the mean ± 1 SEM from two experimental replicates

### CK2α and CK2α' protein levels significantly correlate with oncomir transcript levels in prostate cancer cells by correlation matrix analysis

**Fig. 3:**
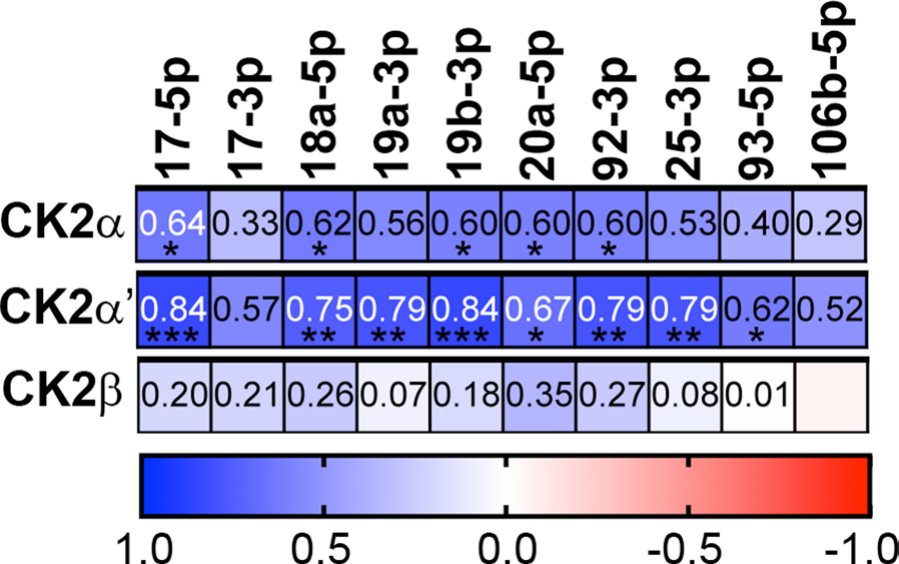
Correlation matrix analysis of CK2 subunit protein levels with miR-17 ~ 92 and miR-106b ~ 25 levels. Correlation analysis of CK2α, CK2α', and CK2β protein with the oncomir cluster miRNA member levels in the 3 prostate cancer cell lines. Bar shown below the matrices indicates correlation gradient from positive (blue) to negative (red) correlation. P values, two-tailed, calculated from Prism 10 Pearson r correlation matrix analysis. *p < 0.05; **p < 0.01, ***p < 0.001

A correlation matrix analysis was performed to compare CK2 subunit protein expression levels with transcript levels of the oncomir cluster members. Combining the data for all prostate cancer cell lines, the normalized values for each CK2 subunit protein in siCK2 and siCtrl samples were analyzed by Pearson r correlation matrix against the normalized values for each of the oncomir miR transcripts. There were significant positive correlations identified for CK2α and highly significant positive correlations identified for CK2α´ with many of the miR-17 ~ 92 and some of the miR-106b ~ 25 constituents (Fig. 3; Table S2). Correlations between CK2β and the miRNA transcripts were not significant. The full correlation matrix data comparing the individual miRNAs is provided in Figure S1.

### CK2 downregulation reduces oncomir expression in breast and head and neck cancer cells

**Fig. 4:**
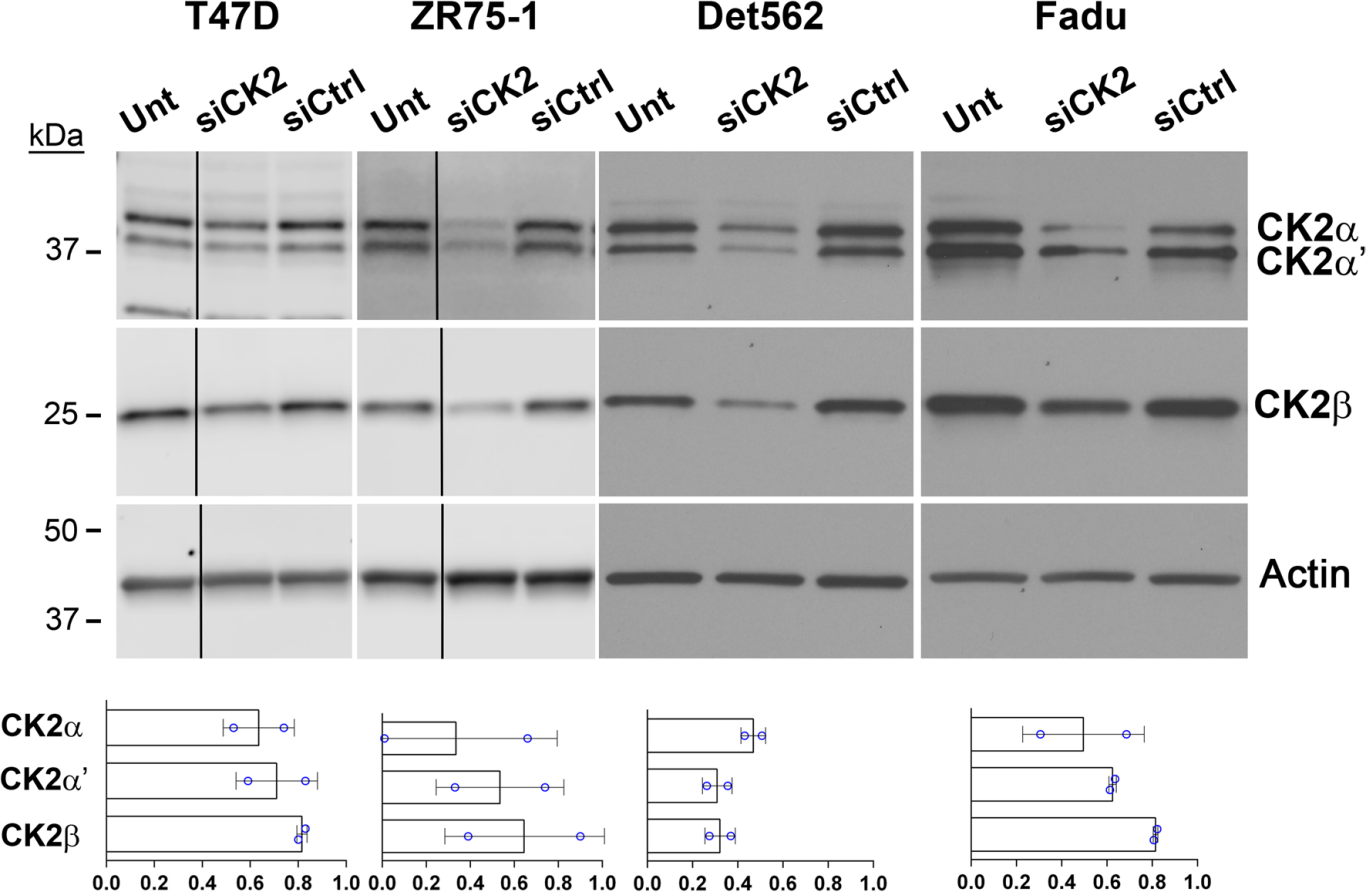
Immunoblot analysis of CK2 downregulation in breast and head and neck cancer cells. Upper panel: Representative immunoblot analysis of T47D and ZR75-1 (breast cancer) and Det562 and Fadu (HNSCC) cells following siRNA transfection (48 h). Proteins detected are indicated on the right side of the blots. Actin signal was used as the loading control. Vertical lines depict removed lanes. Antibodies: CK2α (A300-197A); CK2α΄ (A300-199A); CK2β (sc-46666); Actin (sc-1616). Lower panel: Charts representing quantitation of protein signals relative to siCtrl treatment. Data points from 2 experimental replicates per cell line. siCtrl = siRNA for non-targeting control

We investigated the effect of CK2 downregulation on the oncogenic miRNAs in ER-positive breast cancer and in head and neck squamous cell carcinoma (HNSCC) cell lines. Cells were transfected with siRNAs simultaneously targeting CK2α and CK2α' or with non-targeting siRNAs. Loss of CK2 holoenzyme expression was confirmed by immunoblot (Fig. 4). Concurrent miRNA expression levels from 48 to 72 h post-transfection were determined by qRT-SL-PCR. Again, blocking CK2 expression resulted in reduced levels of miR-17 ~ 92 and miR-106b ~ 25 member miRNAs in all cancer cell lines (Table 5).

**Table 5:** Expression of oncomir clusters in response to CK2 downregulation in breast and head and neck cancer cells

miR-17 ~ 92							
Cell Line	17-5p	17-3p	18a-5p	19a-3p	19b-3p	20a-5p	92a-3p
T47D	0.71 ± 0.14	0.83 ± nd	0.74 ± 0.17	0.73 ± 0.13	0.72 ± 0.13	0.75 ± 0.18	0.67 ± 0.15
ZR75-1	0.51 ± 0.27	0.52 ± nd	0.54 ± 0.30	0.59 ± 0.34	0.58 ± 0.34	0.54 ± 0.22	0.57 ± 0.15
Detroit 562	0.43 ± 0.01	nd	0.39 ± 0.02	0.35 ± 0.07	0.39 ± 0.04	0.45 ± 0.01	0.44 ± 0.08
FaDu	0.42 ± 0.02	nd	0.46 ± 0.04	0.49 ± 0.03	0.53 ± 0.03	0.44 ± 0.01	0.52 ± 0.05

miR-106b ~ 25							
	25-3p	93-5p	106b-3p				
T47D	0.45 ± 0.12	0.30 ± 0.01	0.30 ± 0.05				
ZR75-1	0.61 ± 0.08	0.46 ± 0.19	0.49 ± 0.23				
Detroit 562	0.83 ± 0.16	0.45 ± 0.01	0.58 ± 0.14				
FaDu	0.94 ± 0.06	0.52 ± 0.03	0.74 ± 0.02				

^*^ Cells were transfected with 20 nM siCK2 siRNAs, 48 h. Expression levels were determined using the ∆∆Cq method relative to siCtrl cells. Data shown is the mean ± 1 SEM from 2 experimental replicates (nd denotes data detected in only 1 experiment, or not detected in neither experiment). T47D & ZR75-1, breast cancer. Detroit 562 & FaDu, head and neck cancer

### Decreased pri-miRNA abundance due to CK2 downregulation

We next determined whether there was a change in the levels of pri-miRNA species due to downregulation of CK2 in the three types of cancer under investigation. Downregulation of the pri-miRNA for miR-17 ~ 92 was observed for 5 of the 6 cancer cell lines (Table 6). In contrast, variable levels of pri-miR-25 and pri-miR-106b were observed in breast and head and neck cancer cells (Table 6). We were unable to obtain PCR product for pri-miRNA-93.

**Table 6:** Expression of pri-miRNAs in response to CK2 downregulation in prostate, breast, and head and neck cancer cells

Cell Line	Pri-miRNA detected		
	17 ~ 92	25	106
C4-2	0.53 ± 0.16	0.64 ± 0.06	nd
PC3-LN4	0.56 ± 0.01	0.66 ± 0.04	nd
T47D	nd	1.75 ± 0.21	1.42 ± 0.66
ZR75-1	0.64 ± 0.30	1.51 ± 0.02	1.02 ± 0.16
Detroit 562	0.43 ± 0.04	0.98 ± 0.06	1.22 ± 0.06
FaDu	0.35 ± 0.14	0.87 ± 0.40	0.49 ± 0.10

Cells were transfected with 20 nM siCK2 siRNAs. After 48 to 72 h, expression levels of pri-miRNAs were determined using the ∆∆Cq method relative to siCtrl cells. Data shown is the mean ± 1 SEM from 2 experimental replicates (nd denotes not detected in 1 experimental replicate or not detected). Normalization gene was B2M for prostate and TBP for breast and head and neck. C4-2 & PC3-LN4, prostate cancer. T47D & ZR75-1, breast cancer. Detroit 562 & FaDu, head and neck cancer

Given the reduction in pri-miRNA levels caused by downregulation of CK2, we examined expression of transcription factors reported to regulate transcription of the miR-17 ~ 92 and mir-106b ~ 25 genes. cMYC and E2F-1 are reported to promote transcription and p53 and Rb are reported to suppress transcription of these oncomir genes. We saw very little consistent alteration of any of these factors across the prostate and breast cancer types (Fig. S2). Insufficient samples remained to examine the head and neck cancer cell lines.

### Reduced oncomir levels are observed in orthotopic PCa tumors and mouse serum following CK2 downregulation

**Fig. 5:**
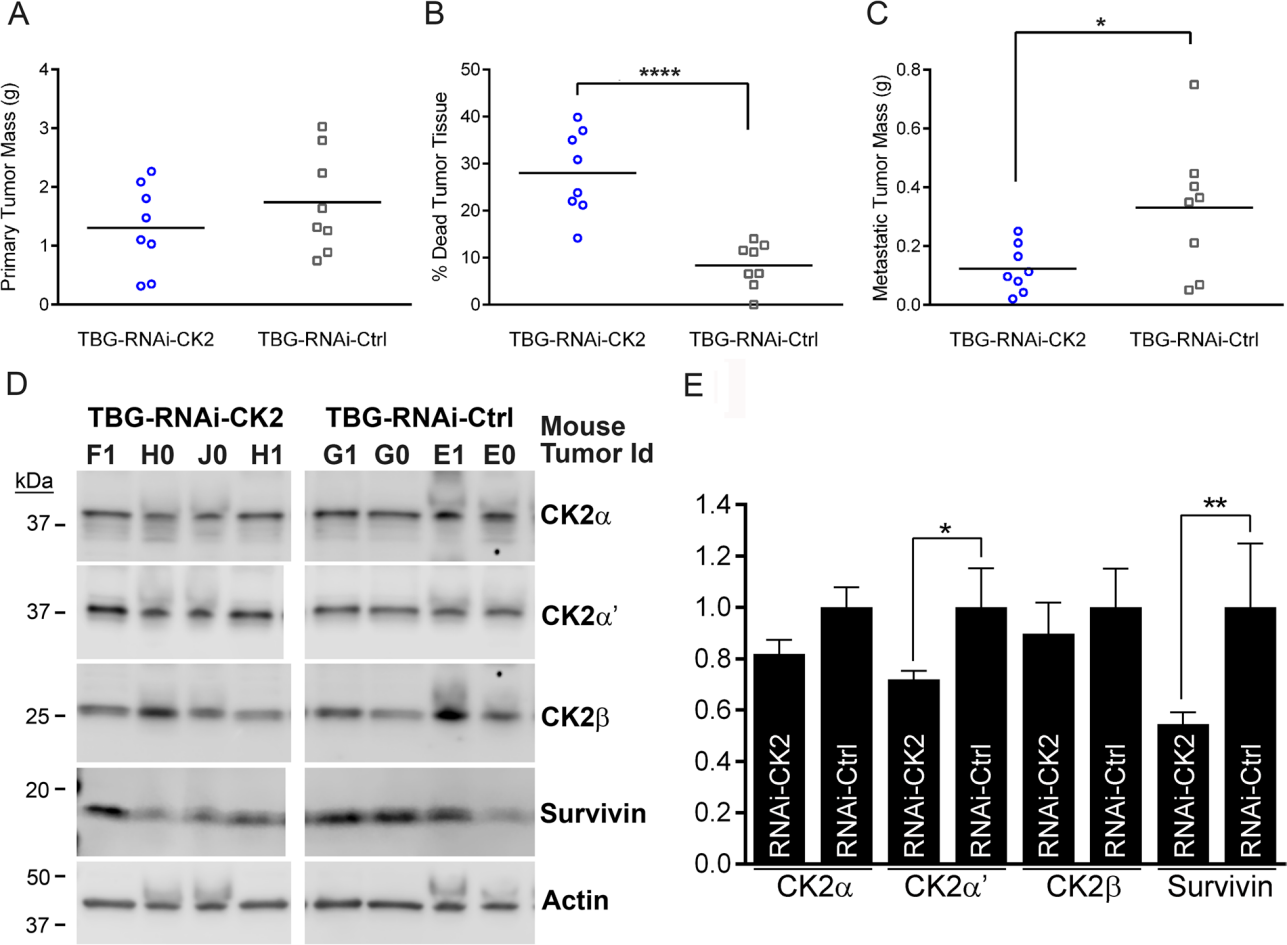
Downregulation of CK2 in PC3-LN4 xenograft tumors. Orthotopic PC3-LN4 tumors were initiated in nude male mice. Following treatment with TBG-RNAi-CK2 or TBG-RNAi-Control (Ctrl) nanocapsule, tumors were harvested, weighed, dissected to remove dead tissue, and reweighed. **A** Tumor mass for each tumor is shown for each mouse. The mean is depicted by the horizontal line. N = 8 per group. **B** Mean percent of dead tumor tissue removed is indicated for each mouse. The mean value is depicted by the horizontal line. N = 8 independent experimental tumors per group. Unpaired t-test, two-tailed. **C** Metastatic tumor mass is shown as sum of all metastatic tumors for each mouse. The mean value is depicted by the horizontal line. N = 8 independent mice per group. Unpaired t-test, two-tailed. **D** Representative immunoblot analysis of CK2 subunit and survivin protein signals in tumor tissue. Proteins detected are indicated on the right side of the blots. Actin signal was used as the loading control. Antibodies: CK2α (A300-197A); CK2α΄ (A1616); CK2β (sc-46666); Survivin (AF886); Actin (sc-1616). **E** Chart representing quantitation of immunoblot data from 4 best response tumors in the TBG-RNAi-CK2 group relative to the TBG-RNAi-Ctrl treatment group (N = 8 controls). Unpaired t-test with Welch’s correction, two-tailed. * p < 0.05; ** p < 0.01; **** p < 0.0001. Error bars represent standard error of the mean (SEM)

Nude mice carrying orthotopic PC3-LN4 tumors were treated with either TBG-RNAi-CK2 or control TBG-RNAi-Ctrl nanocapsule drug. Tenfibgen (TBG), the carboxy-terminal fibrinogen globe domain of tenascin C, has been shown to home to tumors and to deliver anti-CK2 targeted therapies preferentially to tumor cells in xenograft models of human prostate cancer avoiding normal cells (Trembley et al. 2017). The orthotopic tumors grew at disparate rates, which resulted in a large range of tumor sizes and weights for both the anti-CK2 and the control treatment groups. Upon sacrifice, the anti-CK2 treated primary tumors were smaller in mean mass, but this data was not significant (Fig. 5A). However, there was significantly more dead tissue in TBG-RNAi-CK2 treated primary tumors compared to control tumors; these measures indicate that CK2 targeting was successful (Fig. 5B). Additionally, the combined metastatic tumor mass per mouse was significantly reduced for mice treated with the RNAi-CK2 compared to the RNAi-Ctrl nanocapsule (Fig. 5C). We examined these tumors for downregulation of CK2 protein levels as well as impact on Survivin in PCa cells (Fig. 5D). Small reductions in the mean protein expression level were observed for CK2α, CK2α’, CK2β, and Survivin. The charted data for the 4 best responders (those with the highest percentage of dead tissue) are shown relative to control treatment to demonstrate that, although the knockdown of CK2 protein expression in the primary tumors was not significant for all subunits at the time point examined, it was sufficient to downregulate Survivin protein levels similar to observations in cultured cell data (Fig. 5E).

**Fig. 6:**
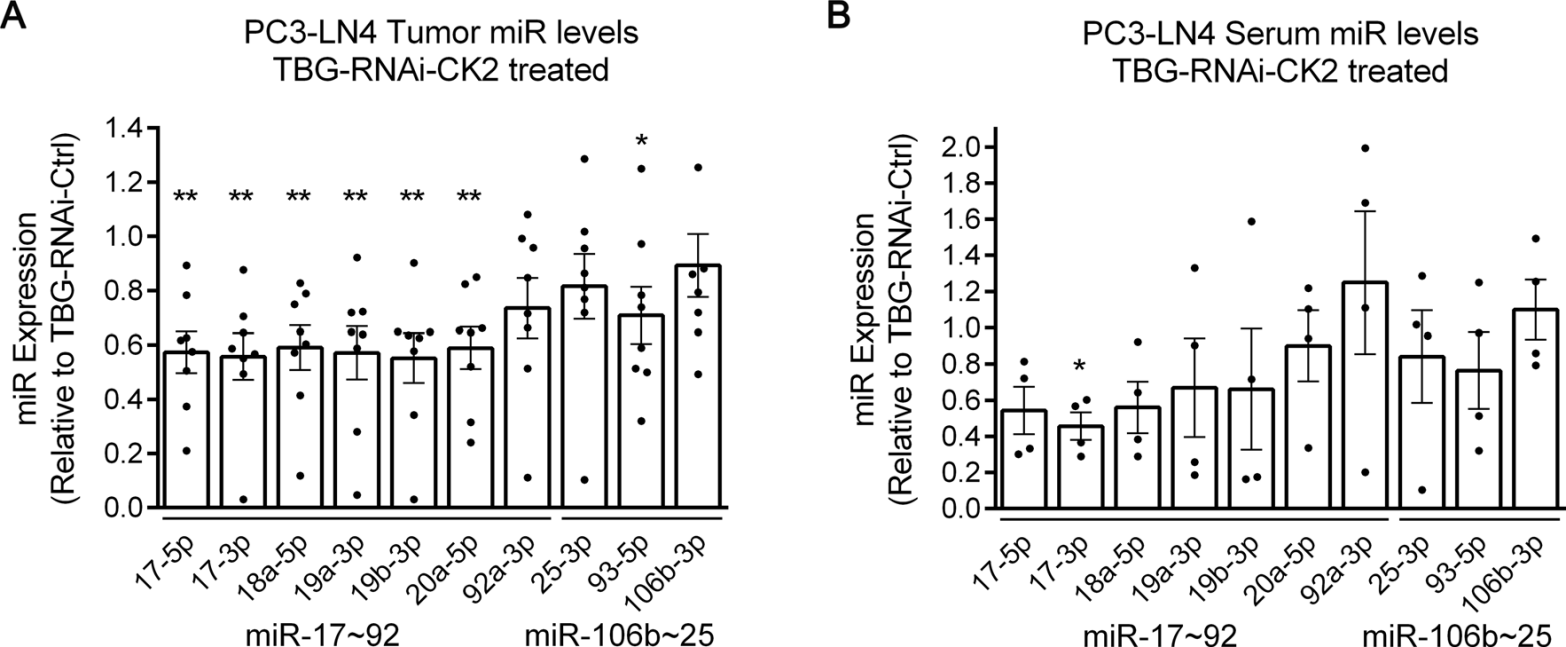
Oncomir expression in PC3-LN4 xenograft tumors and mouse sera. **A** Oncomir q-RT-SL-PCR data in TBG-RNAi-CK2 treatment group tumors relative to TBG-RNAi-Ctrl treatment group. One sample t-test, 2-tailed. **B** Oncomir q-RT-SL-PCR data in TBG-RNAi-CK2 treatment group sera relative to TBG-RNAi-Ctrl treatment group sera. One sample t-test, 2-tailed. *p < 0.05; **p < 0.01

We analyzed miR-17 ~ 92 and miR-106b ~ 25 levels in all primary tumors and determined reduced levels in TBG-RNAi-CK2 treated tumors relative to control tumors; the majority of the changes were statistically significant (Fig. 6A). Next, we also examined miR-17 ~ 92 and miR-106b ~ 25 levels in the serum of these mice. The majority of miR transcripts were similarly reduced in serum compared to what was observed in the tumors; however, because we only had enough serum from groups of 4 mice per treatment condition, statistical significance was only observed for miR-17-3p (Fig. 6B). Thus, our data suggest that CK2 expression is important to maintain intra-tumoral and circulating levels of these mature oncogenic miRNAs.

We accessed data from the CancerMIRNome website to determine whether any of the oncomir cluster miRNA transcripts we examined in mouse serum are known to demonstrate altered circulating levels in human prostate or head and neck cancer (Li et al. 2022). Two miRNAs were identified with significantly increased detection in serum in these cancers relative to non-cancer samples (Table 7).

**Table 7:** Differential detection of oncomir cluster transcripts in human serum in cancer relative to non-cancer

miRNA (mature)	Cancer type	Expression in cancer relative to non-cancer	p value	Adjusted p value
hsa-miR-17-3p	Prostate	5.38	1.59e−203	2.65e−201
hsa-miR-17-3p	Esophageal	3.94	4.34e−18	7.24e−17
hsa-miR-92a-3p	Prostate	6.90	9.19e−02	1.14e−01
hsa-miR-92a-3p	Esophageal	5.50	8.28e−14	6.18e−13

**Fig. 7:**
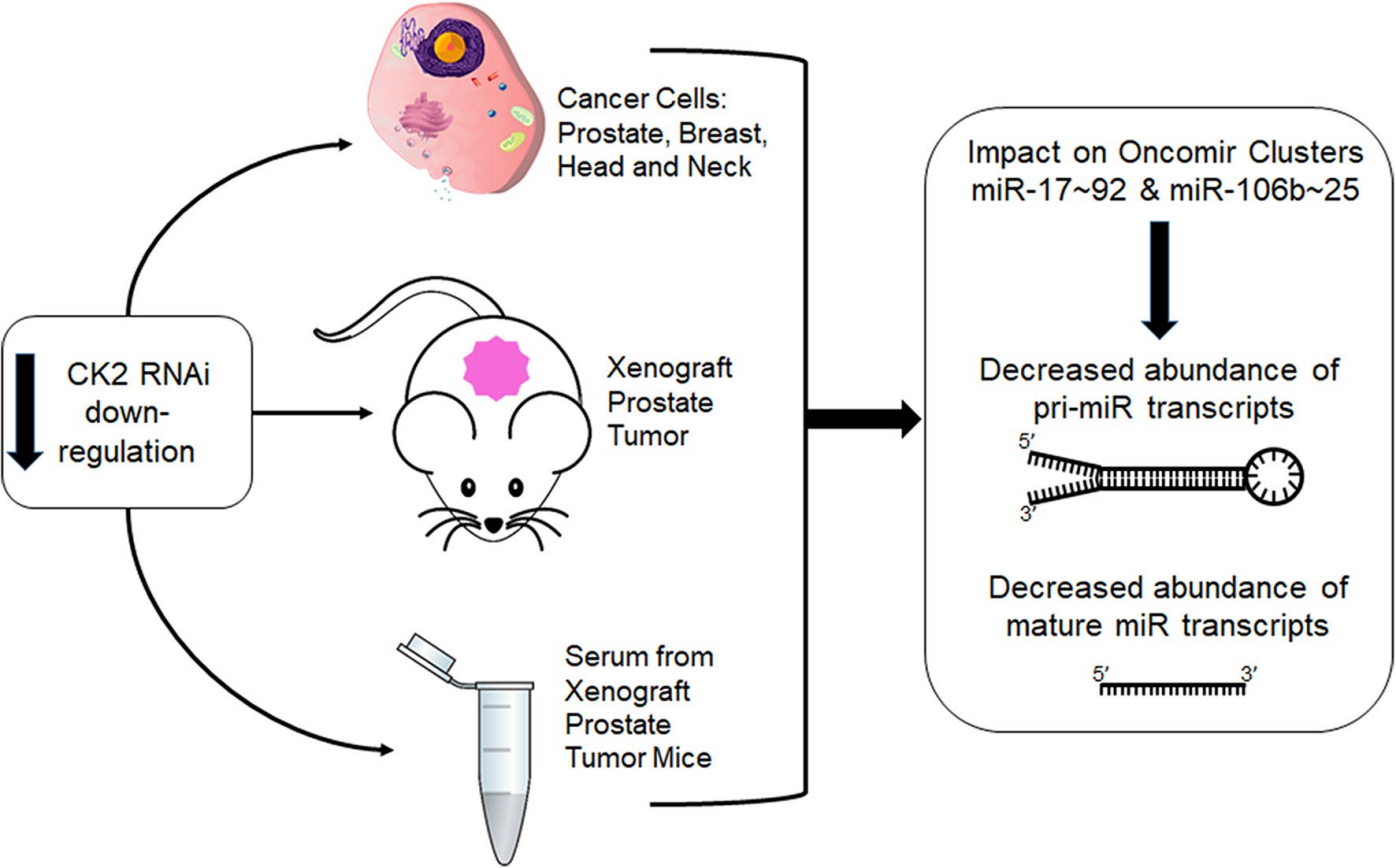
The effects of RNAi-mediated CK2 downregulation in multiple cancer cells and in mice carrying prostate cancer tumors. The cartoon summarizes the key results in prostate, breast, and head and neck cancer cell lines and in prostate cancer xenograft tumors. RNAi-mediated downregulation of CK2 expression levels causes reduction in pri-miRNA and mature miRNA steady state transcript levels in cells, tumors, and blood serum

The data described in this body of work are summarized in Fig. 7.

## Discussion

The complexity of the roles of miRNAs in disease development and progression as well as studies on the regulation of miRNA expression continue to expand (Dragomir et al. 2022). Oncomir clusters 17 ~ 92 and 106b ~ 25 are frequently proposed to play an oncogenic role in cancers, including prostate, breast, and head and neck (Moi et al. 2019; Zhou et al. 2016; Huang et al. 2021). Here we have shown, for the first time, that protein kinase CK2 is involved in regulating the expression of these two oncomir clusters. Blocking CK2 function by both RNAi and small molecule inhibitor methodologies had similar impact on oncomir expression, demonstrating that oncomir loss was not an off-target effect of the chemical inhibitor nor due to engagement of the RNAi/miRNA machinery in human cancer cells. The similar reduction in miRNA levels from the least down-regulated cells and tumors compared to the cells with the highest loss of CK2 suggested that an approximately 20 to 30% loss of CK2 proteins is sufficient to cause reduced oncomir 17 ~ 92 and 106b ~ 25 levels. Interestingly, CK2α´ protein levels were significantly correlated with most and CK2α protein levels with many of the oncomir transcripts evaluated. In contrast, there were no significant correlations between CK2β and the miRNA levels, despite the decreased levels of CK2β caused by siRNA-mediated downregulation of CK2α and CK2α'. This data strongly suggests that the catalytic subunits of CK2 are the key mediators for regulation of expression for these oncomir clusters.

The pri-miRNA data suggested that CK2 functions in cancer cells to support oncomir 17 ~ 92 and 106b ~ 25 expression levels, in part, through mechanisms related to gene transcription. Our examination of the steady-state expression levels for transcription factors known to regulate gene expression for these oncomir clusters was inconclusive. Interpretation of this data is further complicated by the fact that the transcripts for all four of the transcription factor genes are themselves targets of the miR-17 ~ 92 and miR-106b ~ 25 clusters. It is possible that the impact of CK2 downregulation causes changes in protein phosphorylation or other modification status of these or other transcription factors that, in turn, alters protein-DNA interactions, protein–protein interactions, or intracellular localization. In fact, it was recently shown that CK2 participates in the biogenesis of Piwi-interacting RNAs (piRNAs), whereby CK2 activity is necessary for the proper localization and assembly of multiple factors required for piRNA transcription in *C. elegans* (Zhang et al. 2024). The participation of CK2 in a multitude of regulatory networks is well documented (Nuñez de Villavicencio-Diaz et al. 2017).

Emerging data suggest epigenetic regulation of the miR-17 ~ 92 and miR-106b ~ 25 clusters in different tissues and disease states. The miR-106b ~ 25 gene cluster is located within an intron of the MCM7 gene and regulated by a CpG island in the host gene promoter sequence (Morales et al. 2017). The MIR17HG gene, which encodes the miR-17 ~ 92 cluster, has a CpG island 2 kb upstream of its transcription start site (Morales et al. 2017). CK2 is known to phosphorylate and/or interact with several histone deacetylase enzymes (HDACs), as well as lysine-specific demethylase-1 (LSD1), and bromodomain-containing protein 4 (Brd4) (Wu et al. 2013; Pluemsampant et al. 2008; Sun et al. 2002; Costa et al. 2014). CK2 phosphorylation of DNA methyltransferase 3a (Dnmt3a) induced Dnmt3a association with heterochromatin, and CK2 knockdown resulted in genome-wide changes in methylation patterns in repetitive DNA (Deplus et al. 2014). Future investigation into mechanisms for how CK2 sustains oncogenic miRNA expression would include examination of methylation status and histone modifications as well as potential CK2 occupancy of the oncomir genes.

One limitation of this study includes lack of information on the phosphorylation status for CK2-specific sites on transcription factors, in part due to a paucity of reagents. A second limitation is that it is not fully elucidated whether there is differential contribution of individual CK2 subunits (CK2α, CK2α´and CK2β) to the expression of these oncomir clusters. It is unlikely that CK2β knockdown, which was limited in some cell lines and in the tumors and does not significantly correlate with the miRNA transcripts, is acting to affect regulation of the oncomir cluster miRNA levels via an alternate CK2β partner protein; however, this remains as a formal possibility.

## Conclusions and applicability to cancer therapy

Our results show that chemical inhibition or molecular downregulation of CK2 consistently reduced expression of oncomir clusters miR-17 ~ 92 and 106b ~ 25 in prostate, breast, and head and neck cancer cells. The steady-state levels of the CK2 catalytic subunits were significantly and positively correlated with those of the oncomir transcripts in prostate cancer cells. Further, RNAi-targeted reduction of CK2 expression in prostate cancer xenograft tumors reduced the levels of these oncomirs, including intra-tumoral and circulating oncomir transcripts. It is notable that CK2 downregulation significantly reduced the levels of miR-17-3p in mouse serum, and that this oncomir is significantly elevated in serum of patients with prostate cancer. These results have promising implications for incorporating anti-CK2 drugs into treatment strategies to impact tumor signaling and the development of metastatic disease. CK2 has emerged as a druggable target, with several approaches proposed for cancer therapy (Ahmed et al. 2015; Perera et al. 2014; Pierre et al. 2011a; Solares et al. 2009). Effective CK2 targeting using a peptide-based drug was shown in cervical cancer clinical trials (Solares et al. 2009; Sarduy et al. 2015). The CK2 small molecule inhibitor CX-4945/Silmitasertib blocks tumor growth in prostate and other cancers (Pierre et al. 2011b; Siddiqui-Jain et al. 2010). Phase 1 and 2 cancer trials showed that this oral CK2 inhibitor is safe for use with some benefit as a single agent; active single use and drug combination clinical trials continue or were recently completed (NCT03904862; NCT03897036; NCT02128282) (Marschke et al. 2011). These new data demonstrating CK2 function in promoting tumor survival signaling in multiple cancer types highlight the continued clinical promise of anti-CK2 strategies.

## Supplementary Information

Supplementary materials 1.

Supplementary materials 2.

Supplementary materials 3.

Supplementary materials 4.

Supplementary materials 5.

Supplementary materials 6.

Supplementary materials 7.

## Data Availability

Most data generated or analyzed during this study are included in this published article [and its supplementary information files]. Other datasets used and/or analyzed during the current study are publicly available (https://www.cancer.gov/tcga) or available from the corresponding author on reasonable request.

## Abbreviations

CK2: Previous names casein kinase 2 or II
DMSO: Dimethyl sulfoxide
HNSCC: Head and neck squamous cell carcinoma
miR: MicroRNA
miRNA: MicroRNA
oncomir: Onco-genic microRNA
PCa: Prostate cancer
PCR: Polymerase chain reaction
Pri-miRNA: Primary microRNA
RIPA: Radioimmunoprecipitation assay
RISC: RNAi-Induced Silencing Complex
RNAi: RNA interference
RT-PCR: Reverse transcriptase polymerase chain reaction
Ser: Serine
siCK2: Small interfering RNA targeting CK2
siRNA: Small interfering RNA
siNT: Small interfering RNA non-targeting
TBG: Tenfibgen tenfibgen (TBG) which is the carboxy-terminal fibrinogen globe domain of tenascin C
TBB: 4,5,6,7-Tetrabromobenzotriazole
TCGA: The cancer genome atlas
Thr: Threonine
Unt: Untreated
