# Supplementary material for: Impact of protein kinase CK2 downregulation and inhibition on oncomir clusters 17 ~ 92 and 106b ~ 25 in prostate, breast, and head and neck cancers
Source: Mol Med. 2024 Oct 11;30:175. doi: 10.1186/s10020-024-00937-1 (PMC11476306; doi:10.1186/s10020-024-00937-1)
Supplement: Supplementary file 1 — Supplementary materials 1. [file 10020_2024_937_MOESM1_ESM.docx]

**Supplemental Figure Legends**

**Figure S1.** Correlation matrix analysis of CK2 protein and miRNA levels in prostate cancer cells. (**A**) CK2α. (**B**) CK2α'. (**C**) CK2β. Bar to right of each matrix indicates gradient from positive (blue) to negative (red) correlation.

**Figure S2.** Immunoblot analysis of transcription factor expression following CK2 downregulation in prostate and breast cancer cells. Left panels: Immunoblot analysis following siRNA transfection. Proteins detected are indicated on the left side of the blots. Actin signal was used as the loading control on all blots. Antibodies: cMyc (13987); E2F-1 (3742); Rb (sc-102); p53 (1026-1); actin (sc-1616). Right panel: Chart representing quantitation of protein signals relative to si-Ctrl treatment. Data points from 2 or 3 biological experiments (individual data points are shown). siCtrl = siRNA for non-targeting control. * p < 0.05.
