# Supplementary figures and images for: Impact of protein kinase CK2 downregulation and inhibition on oncomir clusters 17 ~ 92 and 106b ~ 25 in prostate, breast, and head and neck cancers
Source: Mol Med. 2024 Oct 11;30:175. doi: 10.1186/s10020-024-00937-1 (PMC11476306; doi:10.1186/s10020-024-00937-1)

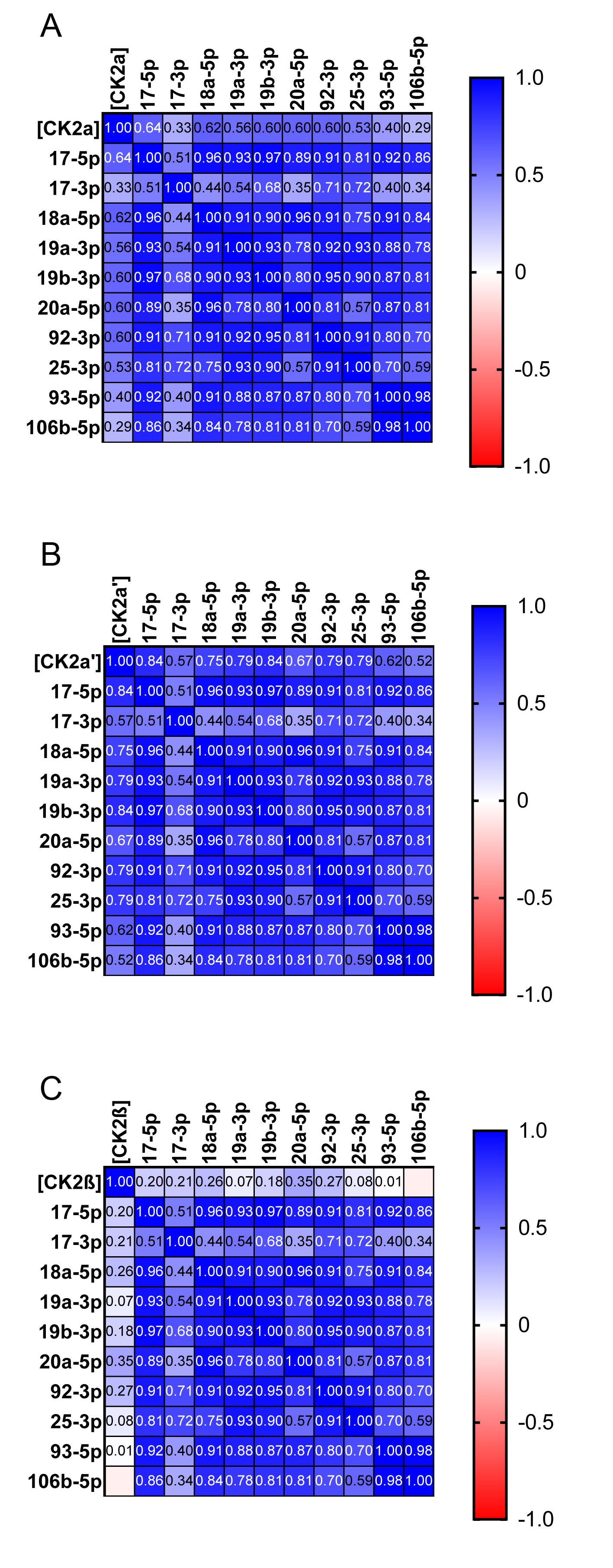

Supplement: Supplementary file 2 — Supplementary materials 2. [file 10020_2024_937_MOESM2_ESM.tif]

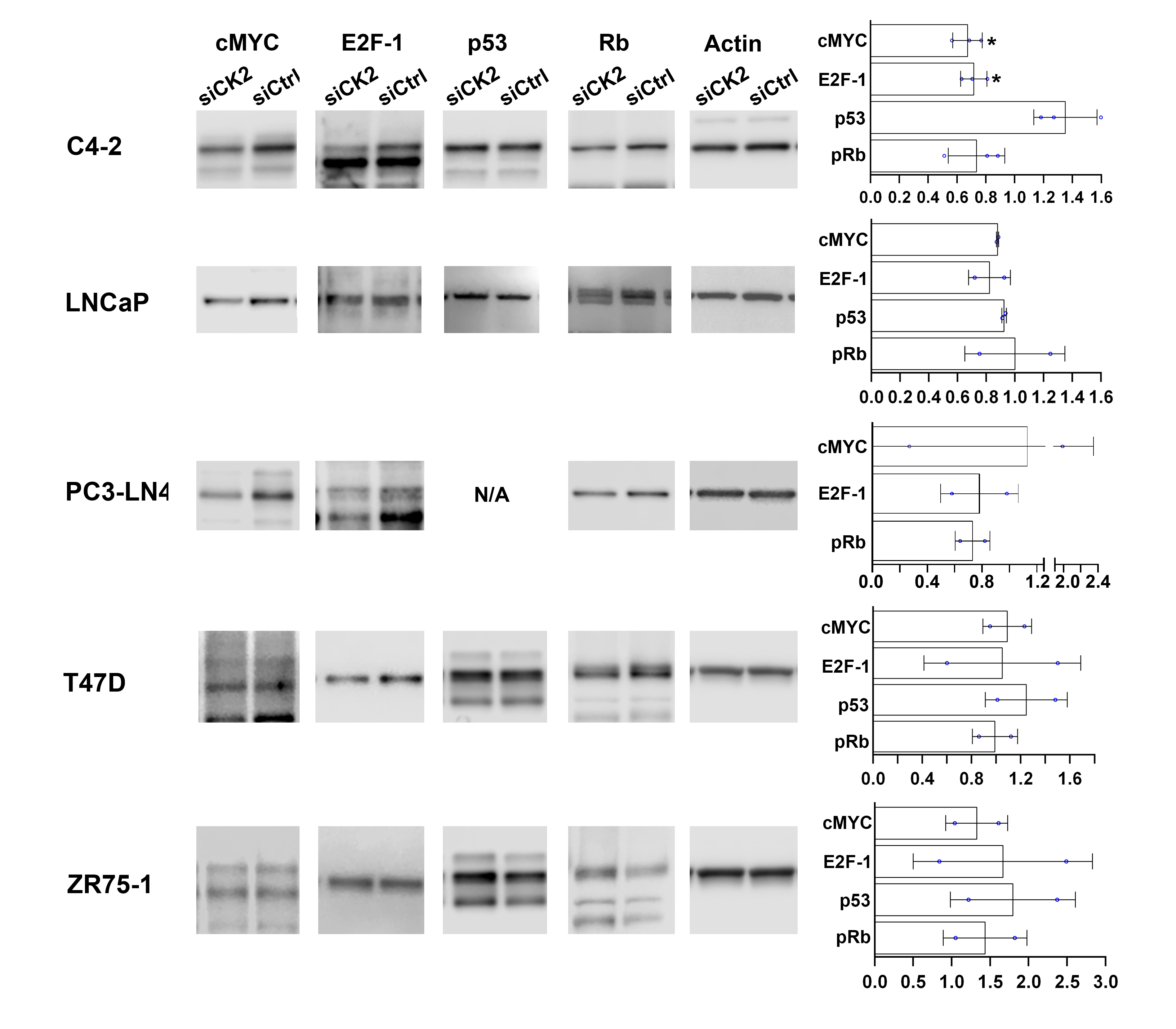

Supplement: Supplementary file 3 — Supplementary materials 3. [file 10020_2024_937_MOESM3_ESM.tif]
